# Letter to the Editor: Detrimental effects of diabetes mellitus in alcoholic liver disease

**DOI:** 10.1097/HC9.0000000000000852

**Published:** 2025-12-01

**Authors:** Muhammad Ali Akbar Syed, Muhammad Abbas, Muhammad Ahsan Asif, Roma Bai, Muhammad Osama Saeed, Sadia Paracha, Saeed Ahmad, Muhammad Tousif Afzal Hafiz

**Affiliations:** Division of Gastroenterology and Hepatology, Beth Israel Deaconess Medical Center, Boston, Massachusetts, USA

We read with great interest the recent article by Ward et al.,^1^ “Diabetes mellitus in alcohol-associated liver disease: Prevalence and outcomes.” This retrospective, multinational study from the WALDO cohort provides valuable insights into the impact of diabetes mellitus on patients with alcohol-associated liver disease (ALD). While this work is timely and has clinical significance, we would like to raise several comments that merit further clarification and discussion.

A strength of this study was the inclusion of patients with biopsy-proven ALD. The authors also discussed the new category of steatotic liver disease, namely, metabolic and alcohol-associated liver disease (MetALD), in the subanalysis. This was an important update, but it was unclear what criteria the authors used to identify MetALD. Key metabolic risk factors, such as high triglycerides, low HDL cholesterol, hypertension, and waist circumference, were not captured. Were only patients with diabetes mellitus considered to have MetALD? In that case, the actual rate of MetALD could be underestimated. Even though the histological findings of metabolic dysfunction–associated steatotic liver disease (MASLD) and ALD are similar, details of biopsy results, such as the degree of steatosis, presence of ballooning degeneration, and Mallory bodies, would enhance the analysis.

In this cohort, 181 patients were reported to have acute alcoholic hepatitis. It was not stated whether liver biopsies were performed during the acute phase; it could have significantly influenced the degree of inflammation and fibrosis. In addition, what was the short-term 90-day mortality of these patients with alcoholic hepatitis with and without diabetes?

The modifications of the natural history of the diseases can directly affect the clinical outcomes. The authors reported that 128 patients discontinued alcohol use at follow-up. That provided an opportunity to compare the prognosis of those with and without alcohol abstinence in addition to the impact of diabetes. Similarly, it would be informative to explore the effects of low-level alcohol intake versus complete abstinence, and how alcohol acts synergistically with diabetes. Equally important, the influence of diabetes management on clinical outcomes needs to be considered. It was unclear what proportion of patients with controlled diabetes by following lifestyle interventions, taking oral hypoglycemic agents, or insulin were included in the study. Notably, patients from the 1970s and 1980s were included; the awareness and management of MASLD and the standard-of-care for diabetes were different from current guidelines. Understanding how alcohol abstinence and optimized diabetes management influence clinical outcomes would provide insights into modifiable risk factors and individualized care.

The cohort enrollment spanned 5 decades from 1971 to 2022; we found it difficult to understand why the median follow-up was only 4.8 years (IQR: 1.2–9.5). The lack of difference in the rates of hepatocellular carcinoma between those with and without diabetes could be due to the relatively short follow-up duration. In a recent publication in the United States, ALD and MASLD were identified as the dominant causes of HCC-associated mortality, currently with an upward trend projected through 2040.^2^ Comprehensive long-term follow-up studies to address the impact of diabetes mellitus on HCC risks among patients with ALD are critical to inform HCC surveillance strategies and policies.

In summary, we congratulate the authors for their important contributions to understanding the roles of diabetes mellitus in ALD. Future studies should provide detailed MetALD criteria, documentation of quantities of alcohol intake and diabetes management, as well as longer follow-up duration to capture HCC incidence. These considerations would shed additional light on this complex and evolving field.
